# Flood protection and endogenous sorting of households: the role of credit constraints

**DOI:** 10.1007/s11027-015-9667-7

**Published:** 2015-08-11

**Authors:** Trond Husby, Henri L. F. de Groot, Marjan W. Hofkes, Tatiana Filatova

**Affiliations:** 1 TNO, Strategy & Policy, Van Mourik Broekmanweg 6, 2628 XE Delft, The Netherlands; 2 Department of Spectral Economics, VU University Amsterdam, De Boelelaan 1105, 1081 HV Amsterdam, The Netherlands; 3 Faculty of Economics and Business Administration and Institute for Environmental Studies, VU University Amsterdam, De Boelelaan 1105, 1081 HV Amsterdam, The Netherlands; 4 Department of Governance and Technology for Sustainability (CSTM), University of Twente, 7500 AE Enschede, the Netherlands; 5 Department of Economics, Scenarios and Innovation, Deltares, Princetonlaan 6-8, 3584 CB Utrecht, the Netherlands

**Keywords:** Flood protection, Climate change, Adaptation, Tiebout-sorting, Credit constraints

## Abstract

Human migration is increasingly seen as a promising climate change adaptation and flood risk reduction strategy. The purpose of this paper is to investigate how spatial differences in flood risk, due to differences in flood protection, reduce the mobility of vulnerable households through a credit constraint mechanism. Using an equilibrium model with two households types and endogenous sorting, we show how spatial differences in flood protection lead to clustering of vulnerable households in a risky region, in a real-world setting of common United States (US) flood zones. We find clustering effects of some size for flood zones with return periods of less than 30 years.

## Introduction

Economic development and rapid urbanisation in coastal regions has led to a sharp increase in the concentration of the world population living in flood-prone areas. Many of these areas are populated by a disproportionate share of vulnerable households. A key challenge for policy makers is to reconcile economic development goals with adaptation goals such as reducing vulnerability in flood-prone areas. Information about the mechanisms that could lead to clustering of vulnerable households in flood-prone areas provides valuable information for the design of adaptation policies.

Human migration out of harm’s way is increasingly seen as a potentially important climate change adaptation option (McLeman and Smith 2006; Black et al. 2011a, b). Climate-driven migration is already pronounced in developing countries, where individual adaptation to increasing risks such as severe floods or droughts is often the only option to avoid risks. Developed countries are usually able to invest in flood protection such as dikes and levees. However, in developed countries, there are still pockets of vulnerable households residing in risky areas. Moreover, as probabilities and severity of climate-related disasters increase, the necessity to activate private adaptation decisions in developed countries grows. A challenge for the design of adaptation policies aimed at stimulating mobility is that household mobility is affected by a range of factors, including the access to mortgage credit. In this paper, we show how spatial differences in flood protection can trigger a credit constraint on vulnerable households, leading to clustering of such households in risky regions.

Flood protection is often considered a public good (Fankhauser et al. 1999). The literature on endogenous Tiebout-sorting suggests that spatial differences in the supply of public goods lead people to vote with their feet, moving to areas that offer a supply in line with their preferences (Tiebout 1956). Low-income households are more likely than high-income households to trade-off public goods against monetary benefits, leading to an increased concentration of low-income households in regions with a low supply of public goods.

As acknowledged by Kuminoff et al. (2010), previous literature has not yet analysed sorting in a setting of natural disaster risk. Much of the sorting-literature has investigated cases where the public good is regulation of currently present nuisance such as air pollution (Banzhaf and Walsh 2008). One obvious challenge for sorting models in the context of flood risk is that people react to risks that they see (Hunter 2005). In the absence of or a long time after large-scale events, low-probability events such as floods may have little direct impact on household decision making (Kunreuther and Pauly 2006). If flood risk is not directly relevant for household preferences, spatial differences in protection will also not be reflected in housing prices. Exogenous shocks causing spatial differences in housing prices would be necessary in order to induce a Tiebout-type of migration among households.

However, the possibility of spatially correlated catastrophic losses means that financial institutions, such as insurance companies and banks, are likely to take flood risk into account (Barnett et al. 2008). Credit providers may wish to shield themselves from covariate risk by raising collateral requirements for mortgages in areas where protection is low. Households who need to take up a mortgage are therefore indirectly affected by flood protection through the credit market. A strand of literature within macroeconomics focuses on how the presence of credit constraints leads to spatial lock-in of households via a housing market mechanism (Quigley 2002; Iacoviello and Neri 2010; Ferreira et al. 2010, 2012). It is precisely this type of mechanism that we exploit to induce the exogenous impulse leading to Tiebout-sorting.

In this paper, we combine the insights from the Tiebout-literature and the macroeconomic literature mentioned above to investigate how spatially differentiated flood protection leads to endogenous sorting of households. Using a general equilibrium model with credit constraints and endogenous sorting, we illustrate how spatial differences in protection lead to clustering of vulnerable households in risky regions. Households in our model choose to locate in the region that offers their preferred combination of economic benefits and disadvantages. The model distinguishes two household types—one which depends on obtaining a mortgage for moving (vulnerable) and one which does not (resilient). In our model, credit lenders do not raise collateral requirements. Instead, the collateral value of a unit of housing is reduced following property valuations carried out by a fictitious real estate agent. These property valuations reflect the probability and housing price impact of a flood. The latter is modelled according to the time-decay function from the hedonic pricing study by Bin and Landry (2013).

The aim of this paper is to show how clustering of vulnerable households in risky regions can occur, in a real-world setting of common United States (US) flood zones. We find clustering effects of some size for flood zones with return periods of less than 30 years. If we assume an average mortgage amortisation period of 30 years, our results suggest that clustering occurs if credit markets can reasonably expect a mortgage holder to have experienced a flood before his or her debt is repaid. It is argued that flood risk reduction strategies should involve soft measures providing stimuli for individual vulnerability reductions (Filatova et al. 2013). Our model provides a connection between housing finance and vulnerability, highlighting the importance of access to mortgage credit for individual vulnerability reductions.

## Literature review

Due to the spatial variation of flood risk and protected economic interests, cost-benefit analysis is increasingly used to inform decisions on flood risk reductions. In order to determine the marginal benefits from protection, economists often try to estimate welfare effects such as willingness to pay for protection. Estimating willingness to pay is complicated by the fact that household demand for public goods such as flood protection is unobservable. To overcome this difficulty, researchers have tried to reveal households’ willingness to pay through their behaviour on the housing market. A number of studies have applied hedonic pricing techniques to analyse the impact of flood risk on housing prices (MacDonald et al. 1987; Speyrer and Ragas 1991; Shultz and Fridgen 2001; Daniel et al. 2009b; Bin and Polasky 2004; Bin et al. 2008; Bin and Landry 2013; Atreya et al. 2013). In a meta-analysis of this literature, Daniel et al. (2009a) find that a 1 percentage point increase in the yearly probability of flooding is associated with a 0.6 % decrease in housing prices.

One drawback of the hedonic pricing approach is that it, by design, depicts a static one-way causal relationship. Charles Tiebout suggested that spatial differences in the supply of public goods could lead people to vote with their feet, moving to areas offering a supply of public goods in line with their preferences (Tiebout 1956). A growing literature of Tiebout-sorting analyses endogeneity effects between the supply of public goods and private decision making (Kuminoff et al. 2010; Kuminoff and Pope 2012; Epple et al. 2010; Sieg et al. 2004). Several authors suggest that interactions between protection and private decision making are important in a flood context as well (Kousky et al. 2006; Hallegatte 2011, 2012). A core element of equilibrium sorting models is how the local supply of public goods leads households to vote with their feet. Migration is driven by the implicit price for public goods, manifested in housing prices, wages or local taxes.^1^Depro et al. (2012) provide evidence of concentration of minorities and low-income households in areas with a high concentration of nuisance. In making a trade-off between lower air quality and more housing services, these households are more likely to take on more air pollution in exchange for a larger house. Banzhaf and Walsh (2008) connect the hedonic-pricing and the Tiebout-literature: if demand for public goods increases with income, lower income households will locate in more risky areas where housing prices are lower. A concentration of low-income households in regions with a low supply of public goods results from a tendency among low-income households to trade off public goods for lower housing costs to a larger degree than high-income households.

Some researchers argue that the decline in housing prices not only works as an attractor but that it also reduces household mobility (Quigley^2002^; Ferreira et al.^2010^, ^2012^): a decline in housing prices reduces housing wealth which again reduces household mobility. A macroeconomic literature investigates the interconnection between credit constraints and household mobility, often focusing on the so-called negative equity problem (The Economist 2010). Chan (2001) finds strong evidence of spatial lock-in due to falling housing prices. Ferreira et al. (2010) and Ferreira et al. (2012) find that negative equity as well as higher mortgage service costs have a large impact on household mobility. Several studies have investigated the labour-market effects of falling housing prices using structural models (Ortalo-Magné and Rady 2006; Iacoviello 2005; Iacoviello and Neri 2010; Nenov 2012; Haavio and Kauppi 2000). The general equilibrium properties of such models allow for investigating feedback effects between falling housing prices and the rest of the economy. Sterk (2010) develops a dynamic stochastic general equilibrium (DSGE)-model, where households face difficulties in moving to a new house as housing prices start to fall. Declining housing prices reduce the collateral value of housing, limiting the possibilities of obtaining a new mortgage. The model predicts that households subject to a debt-constraint suffer a larger reduction in mobility than households who do not rely on a mortgage to finance a move.

## The model

For our analysis, we develop a two-region version of the general equilibrium model from Sterk (2010). The model is defined for two representative households *f*, consisting of a continuum of members, in *t* time periods. We differentiate between a safe and risky region, i.e., *i* = safe,risky. Similar to Sterk (2010), we distinguish between two household types—one type that depends on obtaining a mortgage for financing a move (vulnerable households) and one type that does not (resilient households), i.e. *f* = vulnerable(vul),resilient(re). As mentioned in the Introduction, it is plausible that, in the absence of an event, flood risk has limited direct impacts on households’ decision making. However, demand for housing in risky areas may still be affected if flood risk impacts credit markets. In our version of the model, we separate (endogenous) equilibrium housing prices from (exogenous) property valuations carried out by a fictitious real estate agent. The valuation of risk by credit markets are incorporated in the model through the real estate agent. Property valuations determine the collateral value of a housing unit while equilibrium housing prices affect the demand for housing. Property valuations are therefore only directly relevant for the mobility of vulnerable households.

**Fig. 1:**
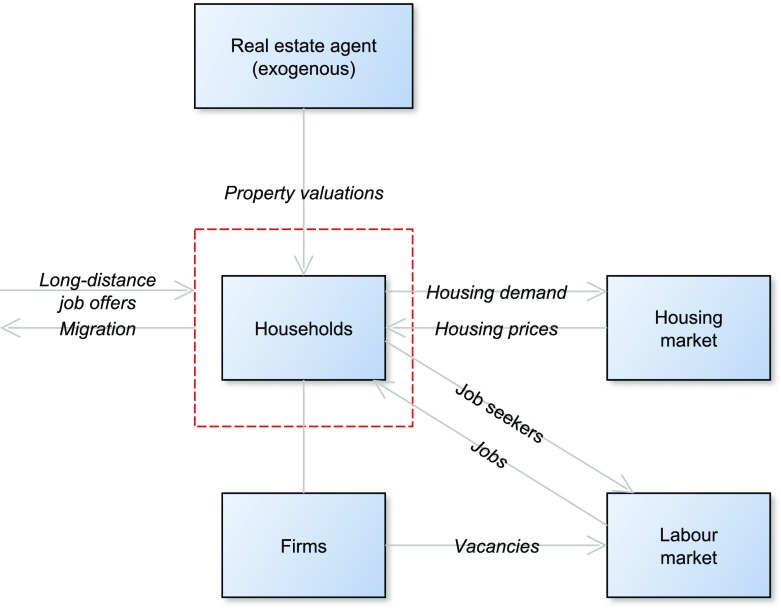
An illustration of how agents within one region interact through the labour and housing markets

Figure 1 illustrates how the agents in a specific region interact through the labour and housing markets. Each region is populated by two representative households and one firm owned by the resilient households. Equilibrium housing prices are determined by equating the (fixed) supply of housing with the aggregate demand. The labour market is modelled according to the Diamond-Mortensen-Pissarides model where firms post vacancies according to profit maximisation and employment is determined by matches between unemployed workers and firms. Long distance job offers can only be accepted if a household moves. A decrease in mobility leads to a rise in unemployment, as households are less likely to move to accept jobs elsewhere.

**Fig. 2:**
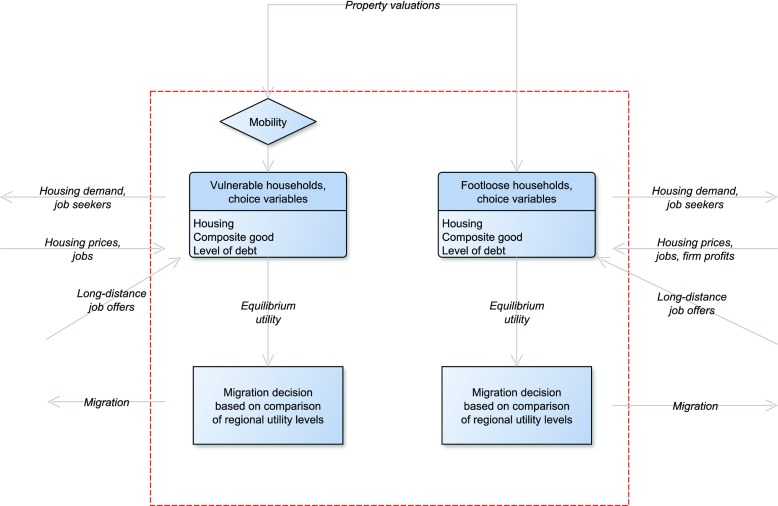
An illustration of how mobility and migration are determined for both household types

Figure 2 illustrates how changes in mobility affect equilibrium utility and the migration decision of the two households. A households’ location decision is modelled as a two-step process, similar in spirit to the push-pull theories of migration (Lee 1966; Massey et al. 1993). In a first step, households evaluate utility at their current location, taking moving costs into account (the mobility decision). This step determines households’ mobility rate. Both aggregate as well as individual factors related to moving away from the current location are taken into account. The first step thus captures the push aspect of migration. In a second step, households compare utility levels across regions (the migration decision). This step corresponds to the pull-aspect of migration, where the attractiveness of different locations is being compared. Vulnerable households depend on obtaining a mortgage to finance a move. Their mobility is therefore tied to the property valuations. Lower property valuations reduce the demand for housing units, leading to a decrease in utility.

Furthermore, lower mobility affects the utility of vulnerable households via two additional channels: it increases the rejection rate of long distance job offers and it reduces expected moving costs. The first channel leads to a decrease in utility, while the latter channel leads to an increase in utility. The mobility of resilient households is not directly influenced by the property valuations as the debt constraint is never binding for this household type. These households are, however, indirectly affected by property valuations through the housing market. The ceteris paribus effect of a reduction in housing demand among vulnerable households implies an increased availability of housing and a decline in the equilibrium housing price. Lower housing prices induce resilient households to purchase the housing units sold by the vulnerable households. The possibility of increased consumption of housing is associated with higher levels of utility, while higher unemployment leads to lower levels of utility.

### Credit markets and flood risk: the real estate agent

Flood risk is incorporated in the model through the property valuations at time *t* in region *i*, *p* *v*_*t*, *i*_, carried out by a real estate agent. The property valuations reflect the concern of credit markets that housing prices can, with a certain probability, deviate from their steady state value due to a flood. In the risky region, we assume, in each time-period, a fixed probability of flooding, while the probability of flooding in the safe region is always zero. The real estate agent evaluates the probability of *one* flood occurring in time-period *t* = 1. Property valuations are carried out in *t* = 0 and are not updated with new housing market information. The real estate agent possesses information about the probability of a flood and about the impact on housing prices, should a flood occur. The probability of flooding is derived from the yearly probability according to return periods of common US flood zones, while impacts from a flood on housing prices are modelled according to the ratio-model in Bin and Landry (2013). This means that *p* *v*_*t*, *i*_ takes on a value lower than the steady-state housing price *p* ^*ss*^ immediately after *t* = 0, converging towards *p* ^*ss*^ over time. The decline in housing prices from a flood is modelled according to the time-decay function in Bin and Landry (2013). Following a standard expected utility formulation, we assume that the expected housing price in time period *t*+1 is a weighted average of housing prices in case of flood and in the case of no flood. The weights used, $Pr_{i}^{rp}$, represent the probability of one flood occurring in time period *t* for various return periods.

Assuming that property valuations are based on the steady-state housing price *p* ^*ss*^, the expected housing price is equal to the steady-state housing price if no flood occurs. Consequently, for the safe region, we have:

1 $$ pv_{t,safe}= p^{ss} $$

Furthermore, we assume a price differential of ${\Delta }^{-}_{t}$ in case of a flood. Consequently for the risky region we have:

2 $$\begin{array}{@{}rcl@{}} pv_{t,risky} &=& (1-Pr_{risky}^{rp}) p^{ss} + Pr_{risky}^{rp} p^{ss}(1+{\Delta}^{-}_{t}) \\ &=& p^{ss}[1+Pr_{risky}^{rp}{\Delta}^{-}_{t}] \end{array} $$

**Fig. 3:**
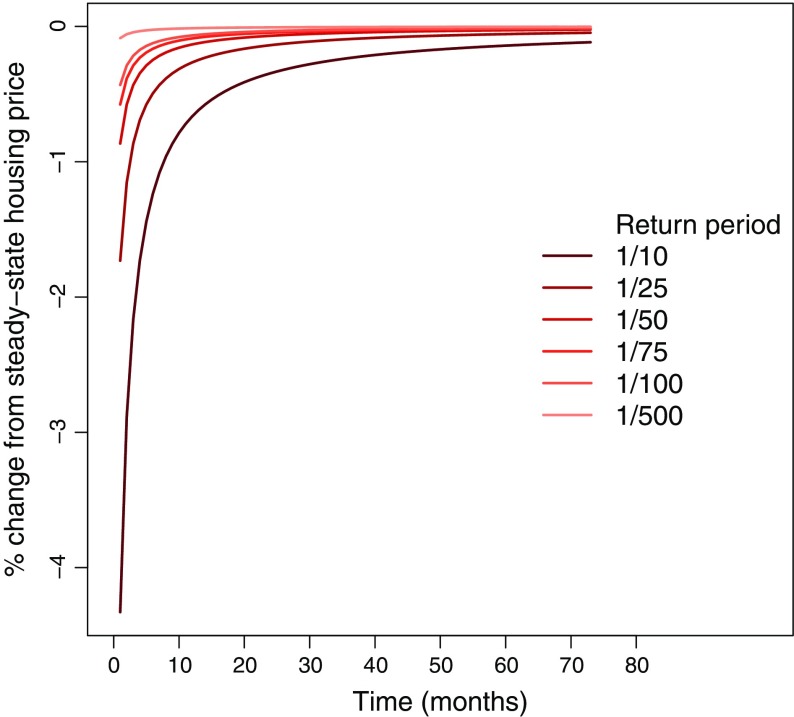
Property valuations carried out by the real estate agent for different return periods (*p* *v*_*t*, *i*_). The horizontal axis shows time-periods (months) after the property valuation, while the vertical axis shows percentage difference from steady-state housing prices

A number of recent hedonic pricing studies have investigated how the housing price effect of a flood decays over time (Bin and Landry 2013; Atreya et al. 2013). Bin and Landry (2013) found a price differential between 6 and 20 %, which essentially disappears after 5–6 years. In order to obtain a *p* *v*_*t*, *i*_ similar to the ratio-model depicted in Bin and Landry (2013, Fig. 2), we specify a functional form for ${\Delta }^{-}_{t}$ which decays at a decreasing rate:

3 $$\begin{array}{@{}rcl@{}} {\Delta}^{-}_{t} = \frac{-{\Theta}}{t} \gamma_{3} \end{array} $$

where *γ*_3_ is the coefficient for the ratio specification in Bin and Landry (2013, Eq. 3). Θ is set such that the difference between the *p* *v*_*t*, *i*_ and the steady-state housing price falls within the range of the estimates from Bin and Landry (2013). Figure 3 plots *p* *v*_*t*, *i*_. We see that its shape corresponds well with the graph in Bin and Landry (2013, Fig. 2). In time period *t* = 1, *p* *v*_*t*, *i*_ is, depending on the return-period, between 0.1 and 4.5 % lower than the steady-state housing price.

### Households: utility and mobility

Let the share of vulnerable households in the economy be denoted by *ν*_*t*, *i*_. Both households receive utility from consumption of non-durable goods *c*_*t*, *i*, *f*_ as well as from square metres of housing *h*_*t*, *i*, *f*_. The fraction of unemployed household members (*n*_*u*, *t*, *i*, *f*_) receive a utility flow *κ*_*f*_ from time spent at home. Households also receive a stochastic locational utility flow *u*_*lo*, *t*, *i*, *f*_. Households choose *c*_*t*, *i*, *f*_, *h*_*t*, *i*, *f*_ and *d*_*t*, *i*, *f*_ so as to maximise utility^2^:

4 $$\begin{array}{@{}rcl@{}} max E_{0}\sum\limits_{t=0}^{\infty} {{\beta_{f}^{t}}}\lbrace ln c_{t,i,f} + \alpha_{f} ln h_{t,i,f} + \kappa_{f} n_{u,t,i,f} + u_{lo,t,i,f} \rbrace \end{array} $$

subject to the budget constraint:

5 $$\begin{array}{@{}rcl@{}} &c_{t,i,f}+p_{t,i}h_{t,i,f}+n_{m,t,i,f}\zeta + R_{t-1}d_{t-1,i,f} \\ &= (1-n_{u,t,i,f})y_{t,i,f}+p_{t,i}h_{t-1,i,f}+d_{t,i,f} \end{array} $$

The left-hand side of the budget constraint shows household expenditures. Here, *p*_*t*, *i*_ is the housing price in units of non-durable goods, *ζ* represents moving costs and *n*_*m*, *t*, *i*, *f*_ is the fraction of moving household members of type *f* at time *t* in region *i* (or, equally, the mobility rate of household type *f*). *R*_*t*−1_ is the gross interest rate on debt from the previous period to be repaid in period *t*, *d*_*t*, *i*, *f*_ is the amount of debt. The right-hand side shows household income. The employed members (1−*n*_*u*, *t*, *i*, *f*_) receive labour-income *y*_*t*, *i*, *f*_. As in Sterk (2010), resilient households receive the profits of the firm in addition to labour income, while vulnerable households only receive labour income. The term *p*_*t*, *i*_ *h*_*t*−1,*i*, *f*_ is the current resale value of the housing stock from the previous period. Income consequently consists of labour-income, new debt and the sales value of the existing housing stock. The amount of debt in each period is restricted by the following constraint:

6 $$\begin{array}{@{}rcl@{}} d_{t,i,f}\leq n_{m,t,i,f}\chi pv_{t,i}h_{t,i,f}+(1-n_{m,t,i,f})d_{t-1,i,f} \end{array} $$

The debt constraint consists of two terms. The first term is only relevant for household members who move. For these household members, a new mortgage will be limited by housing wealth, as assessed by the real estate agent (*p* *v*_*t*, *i*_ *h*_*t*, *i*, *f*_), multiplied by the loan-to-value ratio *χ*. For household members who do not move, the maximum amount of debt is limited by the existing amount of debt (the second term). The fact that the collateral requirement applies only to household members who intend to move, reflects that the debt constraint is really a refinancing constraint.

Households also receive a utility flow from private factors assumed to affect mobility. Such factors include changes in family composition or changes in the neighbourhood. These private factors are incorporated in the model through the locational utility flow *u*_*lo*, *t*, *i*, *f*_. In each period, there is a stochastic shock *𝜖*_*t*, *i*, *f*_. The parameter is normally distributed with mean equal to zero and standard deviation *σ*. In optimum, there will be two cut-off levels for *𝜖*_*t*, *i*, *f*_—one for household members with a long-distance job offer, $\bar {\epsilon }_{do,t,i,f}$ and one for household members without such an offer, $\bar {\epsilon }_{t,i,f}$. The share of household members with a long-distance job offer is *n*_*do*, *t*, *i*, *f*_. Household members that do not move receive *𝜖*_*t*, *i*, *f*_ as a utility flow, while household members that move receive a fixed utility flow *ψ*_*f*_ instead. *ψ*_*f*_ is, as in Sterk (2010), treated as a parameter, and can be interpreted as perceived benefits of moving, before a move has actually taken place.^3^ The presence of *ψ*_*f*_ means that moving is attractive for members with a relatively low realisation of *𝜖*_*t*, *i*, *f*_. If *𝜖*_*t*, *i*, *f*_ is below the cut-off level, the household member will move. *u*_*lo*, *t*, *i*, *f*_ consequently becomes:

7 $$\begin{array}{@{}rcl@{}} u_{lo,t,i,f} &=& n_{do,t,i,f}\left[\psi_{f} F(\bar{\epsilon}_{do,t,i,f})+{\int}_{\bar{\epsilon}_{do,t,i,f}}^{\infty}\epsilon dF(\epsilon)\right] \\ &+&(1-n_{do,t,i,f})\left[\psi_{f} F(\bar{\epsilon}_{t,i,f})+{\int}_{\bar{\epsilon}_{t,i,f}}^{\infty}\epsilon dF(\epsilon)\right] \end{array} $$

Here, *F*(.) represents the cumulative density function of the shock *𝜖*_*t*, *i*, *f*_. *F*(*𝜖*_*do*, *t*, *i*, *f*_) is therefore the mobility rate of household members with a long-distance job offer, while *F*(*𝜖*_*t*, *i*, *f*_) is the mobility rate of household members without such an offer. The mobility rates follow from the utility flow cut-offs:

8 $$\begin{array}{@{}rcl@{}} n_{m,t,i,f} = n_{do,t,i,f}F(\bar{\epsilon}_{do,t,i,f}) + (1-n_{do,t,i,f})F(\bar{\epsilon}_{t,i,f}) \end{array} $$

Declining mobility has two countervailing effects on utility. On the one hand, a decline in mobility leads more household members to refuse long distance job offers, causing an increase in unemployment. Increased unemployment leads to a reduction in utility. On the other hand, lower mobility also entails less expected moving costs, leading to an increase in disposable income and consumption possibilities.

As mentioned above, the resilient households receive the profits of the firm. Profits can be increased by posting less vacancies (see Appendix A). Resilient households thus finance the purchase of housing by posting less vacancies, causing even higher unemployment in the risky region. This assumption may sound ad hoc, but an increase in unemployment in risky regions is consistent with the mechanism envisaged by Kousky et al. (2006), where firms relocate to regions offering higher levels of protection.

### The effect of property valuations on equilibrium housing prices

One of the main innovations of our model relative to Sterk (2010) is the separation between property valuations and equilibrium housing prices. The impact from property valuations on housing prices can be illustrated in Eq. 9 which is the first-order condition for housing (for derivation of all first-order conditions, see Appendix C):

9 $$\begin{array}{@{}rcl@{}} \frac{p_{t,i}}{c_{t,i,g}} = \frac{\alpha_{f}}{h_{t,i,f}} + \beta_{f} \frac{p_{t+1,i}}{c_{t+1,i,f}} + \lambda_{cc,t,i,f}n_{m,t,i,f}\chi pv_{t,i} \end{array} $$

The right-hand side of Eq. 9 shows the shadow-value of housing which indicates the utility change due to an incremental change in *h*_*t*, *i*, *f*_. The shadow-value consists of three terms: the direct utility gain from a marginal unit of housing; the utility gain from the discounted resale value of a unit housing; the additional borrowing capacity generated by an extra unit of housing. *λ*_*cc*, *t*, *i*, *f*_ in this equation is the Lagrange-multiplier on the debt constraint. Since *λ*_*cc*, *t*, *i*, *f*_ = 0 for resilient households, property valuations are not directly relevant for their optimal choice of housing. Equation 9 must be satisfied for both *λ*_*cc*, *t*, *i*, *re*_ = 0 and *λ*_*cc*, *t*, *i*, *vul*_≠0. Housing prices are implicitly determined by the closure of the housing market in Eq. 10, where $\bar {h}_{i}$ is the fixed supply of housing:

10 $$\begin{array}{@{}rcl@{}} \bar{h}_{i} = \nu_{t,i} h_{t,i,vul} + (1-\nu_{t,i}) h_{t,i,re} \end{array} $$

The effect on equilibrium housing prices from a change in property valutions, $\frac {\partial p_{t,i}}{\partial pv_{t,i}}$, cannot be derived analytically, but our simulation results suggest that there is a positive relationship.

### Interregional migration

The variable *n*_*m*, *t*, *i*, *f*_ is referred to as geographical mobility by Sterk (2010). This variable is in optimum determined by a households’ ability and incentives to relocate to another region. In Sterk (2010), there is no actual relocation taking place, meaning that the share of vulnerable households must remain constant. We extend the model in Sterk (2010) by explicitly modelling migration flows. This innovation in the model allows us to endogenously determine the share of vulnerable households in each region.

As a set up for migration decisions, we use a a modified version of the Braun Model of Migration and Growth in Barro and Sala-i-Martin (2003). Population at time *t* in region *i* of household-type *f*, *P* *o* *p*_*t*, *i*, *f*_, is a stock which grows at the rate *γ*_*L*, *t*, *i*, *f*_:

11 $$ Pop_{t,i,f} = Pop_{t-1,i,f}(1+\gamma_{L,t-1,i,f})  $$

We set initial population to *P* *o* *p*_0,*i*, *f*_ = 1. *γ*_*L*, *t*, *i*, *f*_ is defined in line with Barro and Sala-i-Martin (2003, Eq. 9.36) where households compare costs and benefits from moving through a comparison of the benefits from a move with a world-level of benefits. In Barro and Sala-i-Martin (2003, Eq. 9.31), *B*_*t*, *i*, *f*_ represents the net benefits from a move while the world-level of benefits are defined as $B_{t,world,f} = {\sum }_{j} B_{t,i,f}$. The growth rate of the population becomes:

12 $$ \gamma_{L,t,i,f} = \psi_{mig} \left[\frac{B_{t,i,f}}{B_{t,world,f}}\right]  $$

We define the benefits from moving similarly to Barro and Sala-i-Martin (2003). However, while they use the net present value of a wage differential to represent benefits from migration, we use the equilibrium utility, $U_{t,i,f}^{*}$, which is determined by plugging in optimised values of the variables in the utility function. In our two-region setup, the world-level benefits are also not a constant, but determined as the average of the utility in the two regions. As in Barro and Sala-i-Martin (2003), *ψ*_*mig*_ is a function which satisfies the conditions $\psi _{mig}^{\prime }>0$, $\psi _{mig}^{\prime \prime }<0$ and *ψ*_*mig*_(0)=0. We therefore have:

13 $$\begin{array}{@{}rcl@{}} \gamma_{L,t,i,f} = \psi_{mig} \left[\frac{U_{t,i,f}^{*} - U_{t,world,f}^{*}}{U_{t,world,f}^{*}}\right] \end{array} $$

which after some calculations gives:

14 $$\begin{array}{@{}rcl@{}} \gamma_{L,t,i,f} = \psi_{mig} \left[\frac{U_{t,i,f}^{*} - U_{t,j,f}^{*}}{U_{t,i,f}^{*} + U_{t,j,f}^{*}}\right] \end{array} $$

As can be seen from Eq. 14, population growth in region *i* will be positive as long as benefits obtained in that region are larger than those region *j* and negative if in smaller. By setting all choice variables equal to their steady-state values, utility will be equal in both regions, resulting in zero population growth. The two-region setup entails symmetric migration flows between the regions in the sense that emigration from one region is immigration in the other. We model *ψ*_*mig*_ as a sigmoid function to impose such symmetry:

15 $$ \gamma_{L,t,i,f} = \psi_{mig} \left[\frac{(U_{t,i,f}^{*} - U_{t,j,f}^{*})}{U_{t,i,f}^{*} + U_{t,j,f}^{*}}\right] = \psi_{mig}(\hat{B}_{t,i,j,f}) $$

*ψ*_*mig*_ takes the functional form:

16 $$ \psi_{mig}(\hat{B}_{t,i,j,f}) = \frac{1}{1+\exp(-\varphi_{mig}\hat{B}_{t,i,j,f})}-0.5 $$

where *φ*_*mig*_ is a parameter used for calibration. This function satisfies the conditions from Barro and Sala-i-Martin (2003) for $\hat {B}_{t,i,j,f} \geq 0$. For $\hat {B}_{t,i,j,f}<0$, we have $\psi _{mig}^{\prime }<0$ which accounts for the symmetry. The share of vulnerable households in each region is therefore:

17 $$ \nu_{t,i} =\frac{Pop_{t,i,vul}}{{\sum}_{f} Pop_{t,i,f}}  $$

### Welfare effects measured by equivalent variation

To analyse the welfare effects due to the economic changes, we use the equivalent variation (EV), defined as “the gain in income which if experienced without the price falling would make the consumer as much better off as he is made by the fall in price without a change in money income” (Hicks 1943). In our case, we interpret EV as a measure of the additional income necessary for a household to be as well off as if the fall in housing prices had not happened. Normally, a reduction in prices is associated with higher utility levels due to an increase in disposable income, but in our model, the reduction in housing prices also entails a reduction in housing wealth. Our EV measure is calculated by substituting Eq. 5 into the utility function:

18 $$\begin{array}{@{}rcl@{}} U_{t,i,f} &=& ln[(1-n_{u,t,i,f})y_{t,i,f}+d_{t,i,f}-p_{t,i}(h_{t,i,f}-h_{t-1,i,f}) \\ &-& n_{m,t,i,f}\zeta - R_{t-1} d_{t-1,i,f} ] + \alpha \ln h_{t,i,f}+\kappa_{f} n_{u,t,i,f} + u_{lo,t,i,f} \end{array} $$

Equivalent income $y_{t,i,f}^{EV}$ is the income needed to reach $U_{t,i,f}^{*}$, given steady-state values of all other variables in the utility function (18). Steady-state values are denoted with superscript ss to yield:

19 $$\begin{array}{@{}rcl@{}} y_{t,i,f}^{EV} &=& \frac{1}{(1-n_{u,f}^{ss})} \left[ \exp\left( U_{t,i,f}^{*} - \alpha \ln h_{i,f}^{ss} - \kappa_{f} n_{u,i,f}^{ss} - u_{lo,i,f}^{ss} \right) \right. \\ &-&\left.d_{i,f}^{ss}(1-R^{ss}) + n_{m,i,f}^{ss}\zeta \right] \end{array} $$

Our EV measure is the difference between $y_{t,i,f}^{EV}$ and steady-state income $y_{f}^{ss}$, measured as a percentage of steady-state income:

20 $$\begin{array}{@{}rcl@{}} EV_{t,i,f}=\frac{y_{i,f}^{ss} - y_{t,i,f}^{EV} }{y_{i,f}^{ss}} \end{array} $$

### Parameter values and steady-state targeting procedure

The estimation procedure for the parameters reported in Table 1 follows the steady-state targeting strategy and uses the same data as Sterk (2010). This involves setting parameter values such that the model in its steady state reproduces some essential characteristics of the empirical data. Some parameters are also set based on observations from the literature. The data used by Sterk (2010) is US data. Since our parameter values are very similar to those in the original model, we keep the discussion on this point relatively short, reporting only values for the parameters where our values deviate from Sterk (2010). The full list of parameters and the original values can be found in Sterk (2010, Table 3). For a discussion on the parameter values of the labour-market matching function, please consult Sterk (2010, pp. 22–23). Differences in parameter values between our model and (Sterk 2010) are due to the somewhat different set up of our model. However, the differences are so small that they do not affect the main conclusions drawn in this paper.

**Table 1:** Descriptions, values and sources of the parameters used in the model. The lower part of the table contains parameters which are specific to each household type

Parameter	Description	Value		Source
*ν*_0,*i*_	Initial share of vulnerable households	0.5		
Θ	Parameter in loss-function	–23.667		Own calculations
*γ*_3_	Parameter in loss-function	0.439		Bin and Landry (2013)
		Vulnerable hh	Resilient hh	
*β*_*f*_	Discount factor	0.9899	0.9975	Iacoviello and Neri (2010)
*α*_*f*_	Housing preferences	0.250	0.080	Steady state
*ψ*_*f*_	Utility from new location	–7.037	–7.054	Steady state
*κ*_*f*_	Utility from unemployment	–6.639	–6.665	Steady state

Sterk (2010) sets five targets for his steady-state procedure: aggregate unemployment is set at 5 %; aggregate mobility is 0.65 % per month (0.1 % due to long-distance offers); mobile and vulnerable households consume the same amount of housing in steady state; the steady-state value of housing wealth is 140 % of total output; the probability that a vacancy is filled is 0.34. The discount rates for the different household types follow from Iacoviello and Neri (2010), where the discount factor for the resilient households implies a yearly steady-state real interest rate of 3 %. Note that the monthly frequency implies that the discount factor in Table 1 corresponds to monthly discount rates. The values for the discount rates are within the range of those used in the monetary/real business cycle literature (see, e.g., Iacoviello (2005) for a discussion). The preference parameter for housing in the utility function, *α*_*f*_, follows from the steady-state targeting described above. The low value for the resilient households relative to that of vulnerable households is a result of the requirement that both household types consume the same amount of durable goods in the steady state. The utility flow from moving, *ψ*_*f*_, is also found through the steady-state targeting procedure. Note that in our version of the model, this parameter value differs slightly between household types. The same holds true for *κ*_*f*_ which, due to its negative value should be interpreted as stigma from being unemployed. The negative value of *κ*_*f*_ deviates from more standard models where workers and employers are engaged in a Nash bargaining over the surplus from a match. In such models, (a positive) *κ*_*f*_ affects the incentives for a firm of posting vacancies. In the model in Sterk (2010), where workers receive a fixed share of the surplus, *κ*_*f*_ is only relevant in that it affects the incentives for unemployed workers to accept long-distance job offers.

## Results

In this section, we present the results from our simulation exercise. Simulations are carried out by varying flood probability in the risky region according to the probabilities corresponding to the return periods in six common US flood zones: 1/10 (once every 10 years), 1/25, 1/75, 1/50, 1/100, 1/500. Due to the monthly frequency of our model, the monthly probability of flooding becomes $Pr_{i}^{rp} = \frac {1}{10*12}, \frac {1}{25*12} $ and so forth. In the benchmark scenario, we set the probability of flooding equal to zero. All results are presented as percentage deviations from the steady-state values.

### Mobility and unemployment

**Fig. 4:**
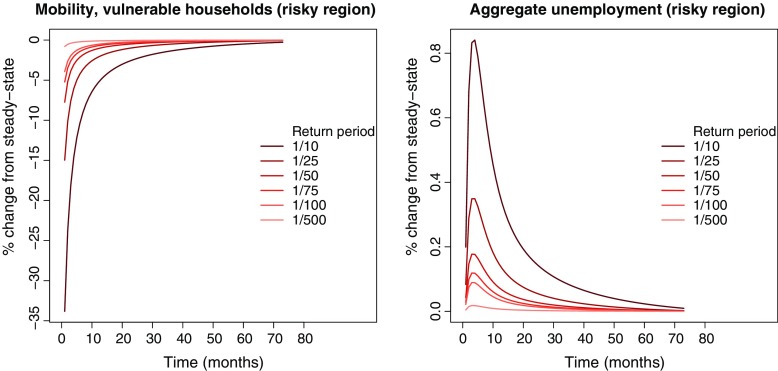
The *left panel* shows the percentage change (relative to steady state) in mobility of vulnerable households in the risky region (*n*_*m*, *t*, *risky*, *vul*_) for different return periods. The *right panel* shows the percentage change (relative to steady state) in aggregate unemployment in the risky region ($\hat {n}_{u,t,risky}$). The horizontal axis shows time-periods (months) after the property valuation

The property valuations *p* *v*_*t*, *i*_ from Eq. 2, illustrated in Fig. 3, limit the maximum debt (see Eq. 6). Only the mobility of vulnerable households is directly affected, as debt is not important for the mobility of resilient households. Comparing Fig. 3 with the left panel of Fig. 4 reveals the direct connection between property valuations and mobility. We readily see that the mobility of vulnerable households follows property valuations, but the relative deviation from steady state for mobility is larger than the relative deviation for property valuations. For the return period 1/10, there is a 35 % immediate reduction in mobility relative to steady state, declining slowly over time. The decrease in mobility leads these households to reject long-distance job offers. This leads to an increase in unemployment as shown in the right panel in Fig. 2. The impact on the mobility of resilient households is only modest since these households are not directly affected by the property valuations. Resilient households also receive the profits of the firm in the economy. In order to compensate for the loss in income due to lower property valuations, resilient households increase firm profits by posting less vacancies leading to a further increase in unemployment.

### Housing consumption and equilibrium housing prices

**Fig. 5:**
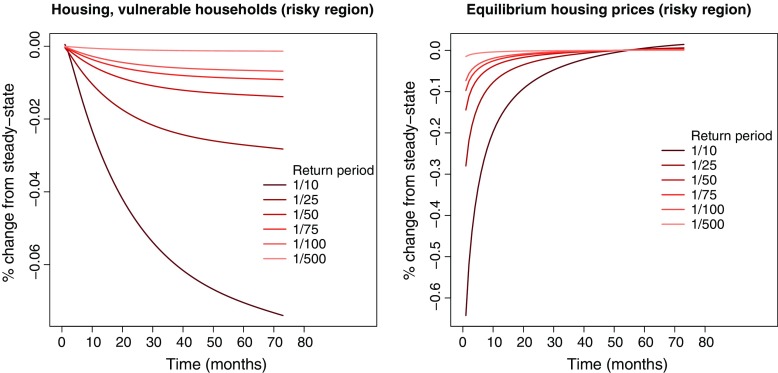
The *left panel* shows the percentage change (relative to steady state) in housing consumption of vulnerable households in the risky region (*h*_*t*, *risky*, *vul*_) for different return periods. The *right panel* shows the percentage change (relative to steady state) in equilibrium housing prices in the risky region (*p*_*t*, *risky*_). The horizontal axis shows time-periods (months) after the property valuation

Lower mobility reduces the incentives for vulnerable households to hold housing stock. This is clearly shown in Fig. 5. We see that the magnitude of the change in housing consumption relative to steady state is limited—at maximum about 0.07 %. It is to be noted that housing consumption returns to its steady state value outside the simulation period. The decrease in demand also reduces the equilibrium housing price (Fig. 5). The decrease in *p*_*t*, *i*_ is similar in shape to *p* *v*_*t*, *i*_, but it returns to the steady-state housing price before *p* *v*_*t*, *i*_ stabilises, and the relative deviation is smaller. The reason behind this result follows from our discussion in Section 3.3. The extent of influence from *p* *v*_*t*, *i*_ on *p*_*t*, *i*_ depends on the Lagrange multiplier *λ*_*cc*, *t*, *i*, *f*_, defined by the Euler equation for debt (Eq. 31 in Appendix B). *λ*_*cc*, *t*, *i*, *f*_ increases initially, dropping steadily over time until it reaches its steady state value around time-period *t* = 59. As vulnerable households shed housing stock, they increase their consumption of non-durable goods over time. In Eq. 31, increased future consumption possibilities reduce the reliance on debt, leading also to a reduction of *λ*_*cc*, *t*, *i*, *f*_. The declining *λ*_*cc*, *t*, *i*, *f*_ dampens the impact from property valuations on equilibrium housing prices.

### Welfare effects and clustering

**Fig. 6:**
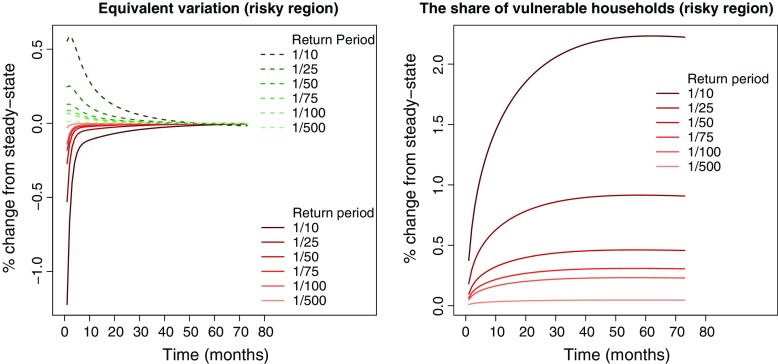
The *left panel* shows the percentage change (relative to the steady state) in equivalent variation for the two households in the risky region (*E* *V*_*t*, *risky*, *f*_). The *solid red lines* indicate vulnerable households while the *dotted green lines* indicate resilient households. The *right panel* shows the percentage change (relative to the steady state) in the share of vulnerable households in the risky region (*ν*_*t*, *risky*_) for different return periods. The horizontal axis shows time-periods (months) after the property valuation

So far, we have identified a clear direct effect of property valuations on the mobility of vulnerable households in the risky region. We also found indirect effects leading firms to leave the risky region, making it less attractive for both household types. This suggests that resilient households would migrate out of the risky region, while vulnerable households remain stuck. The left panel of Fig. 6 reveals a negative EV for vulnerable households, meaning that vulnerable households receive a utility *gain* from property valuations. Our results suggest a utility gain corresponding, at its maximum, to more than 1 % of steady-state income for the return period 1/10. From the discussion in Section 3, we know that lower mobility affects the utility of vulnerable households via two channels: unemployment and moving costs. The negative EV strongly suggests that the moving costs channel dominates. Somewhat paradoxically, it thus appears that vulnerable households experience a welfare *gain* associated with residing in the risky region. However, this is an equilibrium effect originating from the declining mobility of vulnerable households due to the reductions in property valuations. As an increasing share of members of the vulnerable household can no longer afford to migrate, moving costs also decline. The negative EV of the vulnerable household in the risky region should therefore be understood as an equilibrium outcome of reduced mobility for this household type.

The mobility of resilient households is not directly affected by property valuations. However, the deteriorating economic conditions make the risky region unattractive for these households. The utility loss of the resilient households in the risky region related to the increase in unemployment is indicated by the positive EV in the left panel of Fig. 6. As such, for the resilient households increasing unemployment in the risky region is associated with welfare losses, reducing the attractiveness of the region.

The EV results show that vulnerable households find it beneficial to stay in the risky region while resilient households prefer to migrate (EV in the safe region is slightly positive for both household types). Vulnerable households in the safe region will also choose to migrate to the risky region due to the negative EV (higher utility) in this region. The right panel of Fig. 6 shows that the share of vulnerable households for the return period 1/10 is, at its maximum, around 2.2 % higher than the steady state value. The absolute size of the clustering varies only slightly between the return periods 1/500, 1/100, 1/75, and 1/50 (between 50.0 and 51.2 %).

## Discussion

It is widely expected that climate change will lead to sea level rise and to increased river discharge, implying a future rise in flood risk. Reducing exposure and vulnerability are key elements in risk reduction and are consequently high on the climate change adaptation agenda. Migration is increasingly seen as a way of reducing exposure and as an option for climate change adaptation. A key goal in this policy context is to foster household mobility (Findlay 2012). In this paper, we have shown how household mobility decisions may be affected by already existing flood protection measures. Existing protection is likely to result in spatial differences in flood risk. We argue that such differences can lead to a clustering of vulnerable households in risky regions.

Our methodological approach has combined insights from two different strands of literature in a flood risk context, namely the literature on Tiebout-sorting and the macroeconomic literature on spatial lock-in of households via a housing market mechanism. Following the literature of Tiebout-sorting, our model predicts that the supply of a public good (flood protection) affects household behaviour migration decisions via the housing market. However, in line with the psychological literature on decision making under risk and uncertainty, we have assumed that ex-ante risk is not directly relevant for households’ decision making (Slovic 1987; Kunreuther and Pauly 2006). Instead, we have assumed that credit-constrained households are indirectly affected by flood risk if credit markets take risk into account. In our model, we have referred to credit-constrained households as vulnerable, highlighting the effect of lack of credit in a climate change adaptation context. The mobility of these vulnerable households was crucially affected by the property valuations of a fictitious real estate agent. Our results suggest that spatial differences in flood protection lead to clustering of vulnerable households in risky regions. Flood risk not only had a direct impact on mobility but also indirect effects on migration as households reacted to equilibrium price changes. The decline in property valuations in the risky region led to lower demand for housing in this region, causing a difference in housing prices across the two regions. This further incentivised vulnerable households to choose the risky regions.

The research goal of this paper was to investigate how clustering varies in size with the level of protection in the risky region. We only find clustering effects of some magnitude for return periods of 1/10 and 1/25 years. The following back-of-the-envelope calculation illustrates what our results mean in terms of number of inhabitants in the risky region: suppose there is a region with a return period of 1/10 years, with 100,000 inhabitants and an initial share of vulnerable households of 50 %. According to our results, this region would experience an increase in the number of vulnerable households by over 1000. If we assume an average mortgage amortisation period of 30 years, our results suggest that clustering effects can be of concern if credit markets reasonably expect that a mortgage holder will have experienced a flood before the mortgage is repaid.

The mechanisms presented in this paper relate to those discussed by Barnett et al. (2008) who discuss a number of poverty traps where poor households underinvest in productive assets as a manner of reducing future losses. In our model, the vulnerable households are prevented from undertaking ex-ante risk reduction measures (i.e., moving away from hazard before the disaster occurs) due to the presence of a credit constraint. Vulnerable households consequently invest less in assets (housing) due to the reduced collateral services of housing. However, in contrast to Barnett et al. (2008), vulnerable households underinvest in assets as a result of a credit market mechanism and not at as a result of individual risk aversion.

As argued in Husby et al. (2014), a heavy reliance on hard measures in flood risk reduction such as engineered flood defences have in the past led to a concentration of population and economic assets in flood-prone areas. This is the flood risk reduction strategy implemented in a range of developed countries. It is, however, increasingly argued that long-term flood risk reduction strategies should also involve soft measures providing stimuli for individual vulnerability reductions (Filatova et al. 2013). Our model results suggest that facilitating access to mortgage credit could be a potentially important instrument in reducing vulnerability.

Although our model set up and data suggest a developed country context, our results also provide lessons for developing countries. Governments in developing countries often lack the financial resources to fully finance disaster risk, meaning they will have to rely on risk reduction by individual households and on loss sharing through insurance and credit markets. Governments in such countries face challenges with reconciling economic development with adaptation to flood risk. The rapid socio-economic development and rapid urbanisation has led to a dramatic increase in the number of flood-prone areas in these countries. In addition, the increase in the demand for housing has led to changes in the housing finance models of a number of countries such as China and Malaysia, with banks and other private actors gradually replacing the government as the primary provider of mortgages. Our results suggest that such a development could lead to an increased share of vulnerable households in risky areas, which is clearly not in line with adaptation goals.

For the sake of tractability, we have relied on a number of assumptions in our modelling approach. Firstly, the quantitative predictions of our model depend on the value of a number of parameters such as the loan-to-value ratio *χ*. However, as illustrated by the sensitivity analysis in Appendix E, the qualitative predictions of our model appear quite robust. Secondly, our setting is one where there is no actual flood event. We made this assumption to exclude risk as a factor directly influencing household decision making. Abstracting from the impact of an actual flood can also justify the use of expected utility-formulation of the property valuations by the real estate agent. Both of these assumption are important explanations behind the low impacts from flood risk that we find. An extensive literature suggests that decisions made under uncertainty and risk take subjective instead of objective probabilities into account (Kahneman and Tversky 1979; Tversky and Kahneman 1992). Subjective probabilities are particularly relevant in the immediate aftermath of an event (Tversky and Kahneman 1973). Pryce et al. (2011) propose an interesting framework for analysing how housing market responses to floods vary with different types of risk perceptions, without quantifying the effects. An interesting follow-up research to this paper would be an analysis of the quantitative impact of different risk perceptions on housing market outcomes and sorting.

## Appendix A: Labour-market dynamics

In order to keep the description of the model concise, we have left out equations which are defined identically to Sterk (2010) and which are only indirectly relevant for our discussion (e.g., details of the labour market). For a complete list of equations regarding labour market and firms, please consult (Sterk 2010, pp. 16–18). *n*_*d**o*, *t*, *i*, *f*_, the fraction of members with a long-distance job offer, is determined by:

21 $$ n_{do,t,i,f} = \omega \hat{g}_{t-1,i} n_{s,t,i,f}  $$

where $\hat {g}_{t,i}$ is the probability that a firm meets a worker while *ω* represents the fraction of meetings where a worker is required to move in order to become employed. *n*_*s*, *t*, *i*, *f*_, the fraction of household members in search of employment, is defined as:

22 $$ n_{s,t,i,f} = n_{u,t,i,f} + \rho (1-n_{u,t,i,f}) $$

Here, *ρ* is the exogenous job destruction rate. The fraction of unemployed members consists of members who either did not receive a job offer in the previous period or who declined a long-distance offer because moving was unattractive:

23 $$ n_{u,t,i,f} = n_{s,t-1,i,f}(1-\hat{g}_{t-1,i}) + n_{do,t-1,i,f}(1-F(\bar{\epsilon}_{do,t,i,f}))  $$

The aggregate number of households in search of jobs is the share of vulnerable and resilient households searching for jobs

24 $$ \hat{n}_{s,t,i} = \nu_{t,i} n_{s,t,i,vul} + (1-\nu_{t,i}) n_{s,t,i,re} $$

Similarly, aggregate unemployment is:

25 $$ \hat{n}_{u,t,i} = \nu_{t,i} n_{u,t,i,vul} + (1-\nu_{t,i}) n_{u,t,i,re} $$

The labour market is modelled using a matching framework, allowing for labour-market frictions. The aggregate number of matches $\hat {m}_{t,i}$ between firms and job searchers is modelled as a Cobb-Douglas function:

26 $$ \hat{m}_{t,i} = \mu \hat{n}_{s,t,i}^{\eta} \hat{v}_{t,i}^{1-\eta} $$

where $\hat {v}_{t,i}$ is the aggregate number of vacancies, *μ* a scale parameter and *η* an elasticity parameter. The probability that a job searcher meets a firm, $\hat {g}_{t,i}$, is described as:

27 $$ \hat{g}_{t,i} = \frac{\hat{m}_{t,i}}{\hat{n}_{t,i}} $$

In both regions, the representative firm in the economy produces *z*_*a*, *t*, *i*_ per period using labour as input. Households’ labour income is a fixed share *ξ* of total revenues, *y*_*t*, *i*, *f*_ = *ξ*∗*z*_*a*, *t*_, meaning that the firm receives the remaining share (1−*ξ*)*z*_*a*, *t*, *i*_. Aggregate firm profits are therefore given by:

28 $$ \hat{\Pi}_{t,i} = (1-\hat{n}_{u,t,i})(1-\xi)z_{a,t,i} - \vartheta * \hat{v}_{t,i} $$

## Appendix B: Household maximisation problem

The household maximisation problem reads as follows:

29 $$\begin{array}{@{}rcl@{}} max_{c_{t,i,f}, h_{t,i,f}, d_{t,i,f}} E_{0}\sum\limits_{t=0}^{\infty} {{\beta_{f}^{t}}}\lbrace ln c_{t,i,f} + \alpha_{f} ln h_{t,i,f} + \kappa_{f} n_{u,t,i,f} + u_{lo,t,i,f} \rbrace \end{array} $$

s.t.

$$\begin{array}{@{}rcl@{}} &&c_{t,i,f}+p_{t,i}(h_{t,i,f}-h_{t-1,i,f})+n_{m,t,i,f}\zeta + R_{t-1}d_{t-1,i,f} \\ &&= (1-n_{u,t,i,f})y_{t,f}+d_{t,i,f} \\ &&d_{t,i,f}=n_{m,t,i,f}\chi pv_{t,i,f}h_{t,i,f}+(1-n_{m,t,i,f})d_{t-1,i,f} \\ &&n_{s,t,i,f} = n_{u,t,i,f} + \rho (1-n_{u,t,i,f}) \\ &&n_{u,t,i,f} = n_{s,t-1,i,f}(1-\hat{g}_{t-1,i} + \omega \hat{g}_{t-1,i}(1-F(\bar{\epsilon}_{do,t,i,f}))) \\ &&n_{m,t,i,f} = \omega \hat{g}_{r,t-1} n_{s,t,i,f}F(\bar{\epsilon}_{do,t,i,f}) + (1-\omega \hat{g}_{r,t-1} n_{s,t,i,f})F(\bar{\epsilon}_{t,i,f}) \end{array} $$

Maximising the utility function subject to the constraints, we arrive at the following first-order conditions (see also Sterk 2010):

30 $$\begin{array}{@{}rcl@{}} \frac{\alpha_{f}}{h_{t,i,f}} &-& \frac{p_{t,i}}{c_{t,i,g}} + \beta_{f} \frac{p_{t+1,i}}{c_{t+1,i,f}} + \lambda_{cc,t,i,f}n_{m,t,i,f}\chi pv_{t,i} =0 \end{array} $$

31 $$\begin{array}{@{}rcl@{}} \frac{1}{c_{t,i,f}} &-& \lambda_{cc,t,i,f} + \beta_{f}\left[\lambda_{cc,t+1,i,f}(1-n_{m,t+1,i,f}) - \frac{R_{t}}{c_{t+1,i,f}}\right] = 0 \end{array} $$

32 $$\begin{array}{@{}rcl@{}} \frac{\zeta}{c_{t,i,f}} &-& \lambda_{cc,t}(\chi pv_{t,i}h_{t,i,f} - d_{t-1,i,f}) + \bar{\epsilon}_{t,i,f} - \psi_{f} = 0 \end{array} $$

33 $$\begin{array}{@{}rcl@{}} \kappa_{f}&-&\frac{y_{t,i,f}}{c_{t,i,f}} + \bar{\epsilon}_{do,t,i,f} - \bar{\epsilon}_{t,i,f} + (1-\rho_{u})G_{t,i,f} = 0 \end{array} $$

where *λ*_*c**c*, *t*, *i*, *f*_ is the Lagrange-multiplier on the debt constraint, and where *G*_*t*, *i*, *f*_ is a parameter capturing a composition effect on the labour market: if more members with a long-distance offer move, implying an increase in unemployment, positively affecting the fraction of members employed in future periods:

$$\begin{array}{@{}rcl@{}} G_{t,i,f} &=& \left( -\omega \hat{g}_{t-1,i} {\int}_{\bar{\epsilon}_{t+1,i,f}}^{\bar{\epsilon}_{do,t+1,i,f}} \epsilon dF(\epsilon) + (\bar{\epsilon}_{do,t+1,i,f}-\bar{\epsilon}_{t+1,i,f})\left( 1-\hat{g}_{t,i} \right.\right.\\ &+& \left.\phantom{{\int}_{1}^{1}}\left.\omega \hat{g}_{t,i} [1-F(\bar{\epsilon}_{do,t+1,i,f})]\right) + \bar{\epsilon}_{t+1,i,f} \omega \hat{g}_{t,i} [F(\bar{\epsilon}_{do,t+1,i,f})-F(\bar{\epsilon}_{t+1,i,f})]\right) \end{array} $$

It can be shown that:

$$\begin{array}{@{}rcl@{}} {\int}_{\bar{\epsilon}_{t+1,i,f}}^{\bar{\epsilon}_{do,t+1,i,f}} \epsilon dF(\epsilon) = \frac{\sigma}{\sqrt{2\pi}} \left[ \exp\left( -\frac{1}{2} \frac{\bar{\epsilon}_{t+1,i,f}^{2}}{\sigma^{2}}\right) - \exp\left( -\frac{1}{2} \frac{\bar{\epsilon}_{do,t+1,i,f}^{2}}{\sigma^{2}}\right)\right] \end{array} $$

## Appendix C: Description of variables

**Table 2:** Description of the endogenous variables in the model

Variable	Description
*c*_*t*, *i*, *f*_	Consumption of non-durable goods
*h*_*t*, *i*, *f*_	Consumption of housing
*n*_*m*, *t*, *i*, *f*_	Mobility rate
*d*_*t*, *i*, *f*_	Debt
*n*_*s*, *t*, *i*, *f*_	Household job search rate
*n*_*u*, *t*, *i*, *f*_	Household unemployment rate
*n*_*d**o*, *t*, *i*, *f*_	Fraction of household members with a long-distance job offer
*λ*_*c**c*, *t*, *i*, *f*_	Lagrange-multiplier of the debt constraint
*p*_*t*, *i*_	Housing price
*y*_*t*, *i*, *f*_	Labour income
$\hat {g_{t,i}}$	Probability of a labour market match

## Appendix D: Solution procedure and stability of the model

The model in Sterk (2010) is a DSGE model which is solved by the principle of log-linearisation. This approach uses Taylor expansion around the steady state to replace all equations by approximations, where the approximations are linear functions in the log-deviations of the variables. Results from these kind of models generalise to infinite horizon stochastic problems with uncertainty. The model is reformulated as a mixed complementarity problem and programmed in GAMS. This also entails replacing the infinite horizon formulation with a finite time formulation. The approximation of infinite horizon can possibly lead to biased results due to final period effects (Lau et al. 2002). In order to impose stability of the system, we assume zero steady-state growth of all endogenous variables. We furthermore replaced all stochastic parameters from the original model with the mean values of those parameters.

A frequently observed problem with the conversion from infinite horizon to finite time is final period effects. Since a unit of housing has no collateral value in the last period, it could be beneficial for a household to sell an infinitely large amount of housing in the final period, allowing for an infinitely large increase in *c*_*t*, *i*, *f*_. To avoid these kind of effects, we have restricted consumption of all goods to positive values. However, the use of fixed (and modestly sized) discount factors and interest rate, ensures that explosive increases in consumption of non-durable goods are avoided. To see this, consider Eq. 31. For resilient households, for which *λ*_*c**c*, *t*, *i*, *f*_ is always equal to zero, the relation between *c*_*t*, *i*, *f*_ and *c*_*t*+1,*i*, *f*_ is fixed. For vulnerable households, explosive increases in *c*_*t*, *i*, *f*_ are limited by a combination of a fixed discount factor close to 1 and constraints on *λ*_*c**c*, *t*, *i*, *f*_. This result is in line with the Turnpike theorem which states that a competitive equilibrium path converges to a balanced growth path if future consumption is sufficiently weighted. We have experimented with a number of constraints limiting the growth of endogenous variables in the final period, with only minor impacts on our results, confirming the stability of the system and absence of final period effects.

In addition, our model could possibly also suffer from another well known problem in models where households are able to take on debt. One could imagine that a household would like to borrow and consume as much as it likes, paying off interest payments by borrowing more (Ponzi scheme). In our model, debt is irrelevant for equilibrium allocations of resilient households (consider Eq. 32 for *λ*_*c**c*, *t*, *i*, *f*_ equal to zero), eliminating the incentive for this household type to engage in Ponzi schemes. For vulnerable households, the level of debt is controlled by the debt constraint, eliminating the possibilities of ever-increasing levels of debt.

## Appendix E: Sensitivity analysis: varying the loan-to-value ratio

**Table 3:** Impacts on a number of key variables from variations in *χ*. The table shows the original value of *χ* used in the simulation, the original value minus 10 % and the original value plus 10 %

*χ*	0.8			0.72			0.88		
Safety level	1/10	1/100	1/500	1/10	1/100	1/500	1/10	1/100	1/500
*n* *m*_1,*f**l*, *v**u**l*_	−33.83	−3.94	−0.80	−36.75	−4.35	−0.88	−30.82	−3.53	−0.71
*E* *V*_1,*f**l*, *v**u**l*_	−1.22	−0.14	−0.03	−1.36	−0.15	−0.03	−1.09	−0.12	−0.02
*p*_1,*f**l*_	−0.64	−0.07	−0.01	−0.73	−0.08	−0.02	−0.56	−0.06	−0.01
*υ*_20,*f**l*_	1.85	0.20	0.04	2.10	0.23	0.05	1.63	0.18	0.04

## Footnotes

1 See Kuminoff et al. (2010) for a recent review of equilibrium models. For a review of sorting models based on hedonic pricing, see van Duijn and Rouwendal (2012).

2 In Appendix B, the household maximisation problem is presented in more detail. Appendix C provides a list of the endogenous variables in the model. Appendix A gives a description of the labour market dynamics.

3 As in Sterk (2010), *ψ*_*f*_ actually takes on negative values for both household types, which appears unrealistic. However, in the empirical application, what truly matters for the household decision is the difference in distribution between *𝜖*_*t*, *i*, *f*_ and *ψ*_*f*_.

## References

[CR1] Atreya A, Ferreira S, Kriesel W (2013). Forgetting the flood? An analysis of the flood risk discount over time. Land Econ.

[CR2] Banzhaf HS, Walsh RP (2008). Do people vote with their feet? An empirical test of Tiebout’s mechanism. Am Econ Rev.

[CR3] Barnett BJ, Barrett CB, Skees JR (2008). Poverty traps and index-based risk transfer products. World Dev.

[CR4] Barro RJ, Sala-i-Martin X (2003). Economic Growth.

[CR5] Bin O, Landry CE (2013). Changes in implicit flood risk premiums: empirical evidence from the housing market. J Environ Econ Manag.

[CR6] Bin O, Polasky S (2004) Effects of flood hazards on property values: evidence before and after Hurricane Floyd. Land Econ 80(4)

[CR7] Bin O, Kruse JB, Landry CE (2008). Flood hazards, insurance rates, and amenities: evidence from the coastal housing market. J Risk Insur.

[CR8] Black R, Adger WN, Arnell NW, Dercon S, Geddes A, Thomas D (2011a) Migration and global environmental change. Glob Environ Chang 21

[CR9] Black R, Bennett SR, Thomas SM, Beddington JR (2011). Climate change: migration as adaptation. Nature.

[CR10] Chan S (2001). Spatial lock-in: do falling house prices constrain residential mobility. J Urban Econ.

[CR11] Daniel VE, Florax RJGM, Rietveld P (2009). Flooding risk and housing values: an economic assessment of environmental hazard. Ecol Econ.

[CR12] Daniel VE, Florax RJGM, Rietveld P (2009). Floods and residential property values: a hedonic price analysis for the Netherlands. Built Environ.

[CR13] Depro BM, Timmins C, O’Neil M (2012) Meeting urban housing needs: do people really come to the nuisance? Working Paper 18109, National Bureau of Economic Research

[CR14] Epple D, Gordon B, Sieg H (2010). Drs. Muth and Mills meet Dr. Tiebout: integrating location-specific amenities into multi-community equilibrium models. J Reg Sci.

[CR15] Fankhauser S, Smith JB, Tol RSJ (1999). Weathering climate change: some simple rules to guide adaptation decisions. Ecol Econ.

[CR16] Ferreira F, Gyourko J, Tracy J (2010). Housing busts and household mobility. J Urban Econ.

[CR17] Ferreira F, Gyourko J, Tracy J (2012) Housing busts and household mobility: an update. Economic Policy Review (Nov):1–15

[CR18] Filatova T, Verburg PH, Parker DC, Stannard CA (2013). Spatial agent-based models for socio-ecological systems: challenges and prospects. Environ Model Softw.

[CR19] Findlay AM (2012). Migration: flooding and the scale of migration. Nat Clim Chang.

[CR20] Haavio M, Kauppi H (2000) Housing markets, liquidity constraints and labor mobility. Bank of Finland Discussion Papers 8

[CR21] Hallegatte S (2011) How economic growth and rational decisions can make disaster losses grow faster than wealth. Policy Research Working Paper Series 5617, The World Bank, Washington DC

[CR22] Hallegatte S (2012) An exploration of the link between development, economic growth, and natural risk. Policy Research Working Paper Series 6216, The World Bank, Washington DC

[CR23] Hicks JR (1943). The four consumer’s surpluses. Rev Econ Stud.

[CR24] Hunter LM (2005). Migration and environmental hazards. Popul Environ.

[CR25] Husby TG, de Groot HLF, Hofkes MW, Dröes MI (2014). Do floods have permanent effects? Evidence from the Netherlands. J Reg Sci.

[CR26] Iacoviello M (2005). House prices, borrowing constraints, and monetary policy in the business cycle. Am Econ Rev.

[CR27] Iacoviello M, Neri S (2010). Housing market spillovers: evidence from an estimated DSGE model. American Economic Journal: Macroeconomics.

[CR28] Kahneman D, Tversky A (1979). Prospect Theory: an analysis of decision under risk. Econometrica.

[CR29] Kousky C, Luttmer EFP, Zeckhauser RJ (2006). Private investment and government protection. J Risk Uncertain.

[CR30] Kuminoff NV, Pope JC (2012) Do ”capitalization effects” for public goods reveal the public’s willingness to payUnpublished manuscript, Arizona State University, May

[CR31] Kuminoff NV, Smith VK, Timmins C (2010) The new economics of equilibrium sorting and its transformational role for policy evaluation. Working Paper 16349, National Bureau of Economic Research

[CR32] Kunreuther H, Pauly M (2006). Rules rather than discretion: lessons from Hurricane Katrina. J Risk Uncertain.

[CR33] Lau MI, Pahlke A, Rutherford TF (2002). Approximating infinite-horizon models in a complementarity format: a primer in dynamic general equilibrium analysis. J Econ Dyn Control.

[CR34] Lee ES (1966). A theory of migration. Demography.

[CR35] MacDonald DN, Murdoch JC, White HL (1987). Uncertain hazards, insurance, and consumer choice: evidence from housing markets. Land Econ.

[CR36] Massey DS, Arango J, Hugo G, Kouaouci A, Pellegrino A, Taylor JE (1993). Theories of international migration: a review and appraisal. Popul Dev Rev.

[CR37] McLeman R, Smith B (2006). Migration as an adaptation to climate change. Clim Chang.

[CR38] Nenov P (2012) Labor market and regional reallocation effects of housing busts. Job market paper, Massachusetts Institute of Technology

[CR39] Ortalo-Magné F, Rady S (2006). Housing market dynamics: on the contribution of income shocks and credit constraints. Rev Econ Stud.

[CR40] Pryce G, Chen Y, Galster G (2011) Theoretical foundations for understanding the impact of floods on house prices: an imperfect information approach with amnesia and myopia. Hous Stud 26(2)

[CR41] Quigley JM (2002) Homeowner Mobility and Mortgage Interest Rates: New Evidence from the 1990s. Berkeley Program on Housing and Urban Policy, Working Paper Series qt9192767g, Berkeley Program on Housing and Urban Policy

[CR42] Shultz SD, Fridgen PM (2001). Floodplains and housing values: implications for flood mitigation projects. J Am Water Resour Assoc.

[CR43] Sieg H, Smith VK, Banzhaf HS, Walsh R (2004). Estimating the general equilibrium benefits of large changes in spatially delineated public goods. Int Econ Rev.

[CR44] Slovic P (1987). Perception of risk. Science.

[CR45] Speyrer JF, Ragas WR (1991). Housing prices and flood risk: an examination using spline regression. J Real Estate Financ Econ.

[CR46] Sterk V (2010) Home equity, mobility, and macroeconomic fluctuations. DNB Working Papers 265, Netherlands Central Bank, Amsterdam

[CR47] The Economist (2010) Drowning or waving. Available at http://www.economist.com/node/17305544

[CR48] Tiebout CM (1956). A pure theory of local expenditures. J Polit Econ.

[CR49] Tversky A, Kahneman D (1973). Availability: a heuristic for judging frequency and probability. Cogn Psychol.

[CR50] Tversky A, Kahneman D (1992). Advances in Prospect Theory: cumulative representation of uncertainty. J Risk Uncertain.

[CR51] van Duijn M, Rouwendal J (2012). Analysis of household location behaviour, local amenities and house prices in a sorting framework. J Prop Res.

